# Text messaging to improve uptake of human papillomavirus vaccination: a study among adolescent girls living with HIV in Kisumu county

**DOI:** 10.3332/ecancer.2053

**Published:** 2025-12-02

**Authors:** Ochomo Edwin Onyango, Philiph Tonui, Peter Itsura, Elkanah Omenge Orang'o, Kapten Muthoka, Sayo Loice, Benard Ochieng Samba, Barry Rosen, Patrick Loehrer, Susan Cu-Uvin

**Affiliations:** 1 AMPATH, PO Box 4606, Eldoret 30100, Kenya; 2 KEMRI – RCTP, PO Box 614, Kisumu 40100, Kenya; 3 Moi University School of Medicine, PO Box 4606, Eldoret 30100, Kenya; 4 Main-Analytics GmbH, 65843 Sulzbach, Germany; 5 Division of Gynecologic Oncology, Oakland University William Beaumont, Rochester Hills, MI, USA; 6 Indiana University School of Medicine, Indianapolis, IN, USA; 7 Brown University, Providence, RI, USA

**Keywords:** adolescent, HIV, HPV, SMS, vaccination

## Abstract

**Background:**

Despite the availability of human papillomavirus (HPV) vaccine, its uptake remains sub-optimal across all the eligible age groups in sub-Saharan Africa countries. The vaccine hesitancy is driven partly by a lack of information regarding the safety and efficacy of the HPV vaccine. This study evaluated the use of text messaging to improve HPV vaccine uptake in most at-risk population of adolescent girls living with HIV.

**Methodology:**

We enrolled 152 vaccine naïve adolescent girls and randomised them to either an intervention or a control arm. The intervention arm received weekly messages containing information about cervical cancer, HPV and HPV vaccination. The participants were follow-up for 6 months, with their vaccination status recorded at every clinic visit. The difference in the vaccination rates between the intervention and the control arms of the study was analysed using t-test to determine statistical significance.

**Results:**

Of the 151 participants who completed the study, 35 (23.2%) received the first dose of the HPV vaccine by the time the study closed at 6 months. Among these, 9 (25.7%) were respondents in the control arm and 26 (74.3%) in the intervention arm. This difference in HPV vaccine uptake was statistically significant *p* = 0.001. A Kaplan-Meier plot showed a shorter time to vaccination in the intervention arm compared to the control arm.

**Conclusion:**

The use of short text messaging services is a viable communication channel for sharing information about HPV and HPV vaccination with both parents and adolescent girls. This approach has the potential to improve uptake of HPV vaccination.

## Background

The global cervical cancer burden was reported to include 662,301 new cases and 348,874 deaths in 2022 [1], an increase from the 604,127 cases and 341,831 deaths recorded in 2020 [2]. This burden is significantly higher in the low and middle-income countries (LMICs) largely, where preventive services such as vaccination against human papillomavirus (HPV) and diagnosis of precancerous and early-stage cervical cancer through screening are still not properly entrenched in routine healthcare delivery. The low uptake of HPV vaccine is due to factors like high costs of the vaccine, stakeholder resistance due to misinformation [3, 4]. Despite the Global Alliance for Vaccines and Immunisation (GAVI) providing financial support to make the vaccine accessible, challenges still exist, including lack of political good-will and fragmented policy actors, and weak health systems [3].

Many of the developing countries also experience a high prevalence of HIV, which enhances HPV-induced carcinogenesis that puts women in sub-Saharan Africa at a greater risk [5]. HIV infection has also been shown to reduce the efficacy of vaccines, including the HPV vaccine, especially in women and adolescent girls [6]. These two factors underscore the need to focus on this special category of population who, if not vaccinated and protected, are at an elevated risk of developing cervical cancer as they get into adulthood.

Vaccination rates in LMICs indicate that only 15% of girls in the target age group have received the vaccine, with LMICs recording the lowest uptake rates globally [7]. Similarly, a systematic review analysing HPV vaccine uptake in LMICs reported a pooled uptake rate of just 4.72% among the female target population [8]. Vaccination against HPV has been shown to be effective against HPV infection, which increases the risk of developing cancer of the cervix [9, 10], preventing up-to 90% of HPV infections [11, 12], as evidenced by countries where the HPV vaccine has been incorporated into routine immunisation programs.

In the United States, HPV vaccine uptake for at least one dose of the HPV vaccine improved between 2019 and 2020 from 71.5% to 75.1% [13], contributing to the reduction in the number of cervical cancer cases. Similarly, reports from other countries like Rwanda and Bhutan indicate that the vaccine is effective [14]. A trend analysis for national HPV vaccination and coverage rates in Africa shows that countries in the East African region, like Ethiopia, Rwanda, Uganda and Tanzania have made significant progress in the uptake of the first dose of the vaccine ranging from 86% to 73% with a wait time of between 5 and 12 years through the support of GAVI [3]. Concerningly, Kenya has a first dose coverage of only 29% with a waiting time of 13 years [3].

In Kenya, where HPV vaccine is available in a 2-dose schedule for girls aged 9–14 years [15], report from the HPV information centre shows that, the vaccine uptake remains low, with only 33% of the girls receiving the first dose and a completion rate of just 16% [16]. A review by Karanja-Chege [17] reported a pooled vaccination rate of 25%. Further, data from four pilot counties in Kenya in 2020 shows Kisumu lagging behind in both the uptake of the first and second HPV doses [18].

Use of technology has been shown to have a great potential in improving service uptake in various segments of the population [19, 20]. However, among adolescents, this could be hindered due to lack of access to phones, technological literacy, inferior network coverage and legislative barriers to phone ownership [21, 22]. Lack of information is one of the major barriers to vaccine uptake, as reported by the adolescent girls [23]. Despite the parents being the overall decision maker when it comes to vaccination, the role of informed adolescents in influencing the parents/guardians to have them vaccinated cannot be over emphasised [24, 25]. Communication about vaccination should target adolescent girls who are the primary beneficiaries, in a culturally sensitive and age-appropriate manner, to improve awareness and acceptance. The objective of this study was to evaluate the effectiveness of short message services (SMS) in improving the uptake of HPV vaccine amongst adolescent girls living with HIV in Kisumu County, Kenya.

## Methods

### Study area

The study was conducted in Kisumu County, Kenya, among adolescent girls enrolled at the comprehensive care clinics (CCCs) in Kisumu County Referral Hospital (KCRH) and Lumumba Sub-County Hospital (LSCH). The county has seven sub-Counties; Nyando, Nyakach, Seme, Kisumu East, West and Central, and Muhoroni but the main CCC and HPV vaccination centres are Lumumba, and KCRH. According to NASCOP report, the HIV prevalence of the county is 17.3%, ranking second in the country [26].

### Study design and procedures

This study adopted a longitudinal study design, collecting baseline and endline data to assess the HPV vaccine uptake. The intervention involved sending weekly text messages with information about HPV, HPV vaccination and cervical cancer to the adolescent girls in the intervention arm over 6 months. These weekly messages were shared with the adolescents through the guardian’s contacts.

Using the sample size calculation formula by Prasad [27] for two equal-sized groups with dichotomous outcome. A total of 152 unvaccinated girls aged 10–14 years, enrolled at two clinics and were randomised into intervention and control arms through simple randomisation. The participants were enrolled by a research assistant then assigned participant identifiers; through simple randomisation, the lead researcher allocated the participants into the study arms. The participants were blinded and did not know which arms they belonged to. All the study participants received health messages on medication adherence, nutrition, hygiene and corona virus on a weekly basis. However, the intervention arm received additional weekly messages with information about cervical cancer, HPV, HPV vaccine and vaccination and cancer of the cervix. These messages varied weekly to cover different aspects of the topics.

Participants in both the intervention and control arms were followed up for a period of 6 months. During the 6-month study period, they received text messages irrespective of a change in the vaccination status. The vaccination status was updated at each monthly clinic visit.

During the monthly clinic visits, research assistants confirmed that the messages reached the intended recipients by asking the participants and documenting how many messages the guardians or parents had shared with them. For those who reported to have been vaccinated, the research assistant recorded the date of vaccination.

## Results

**Figure 1.**
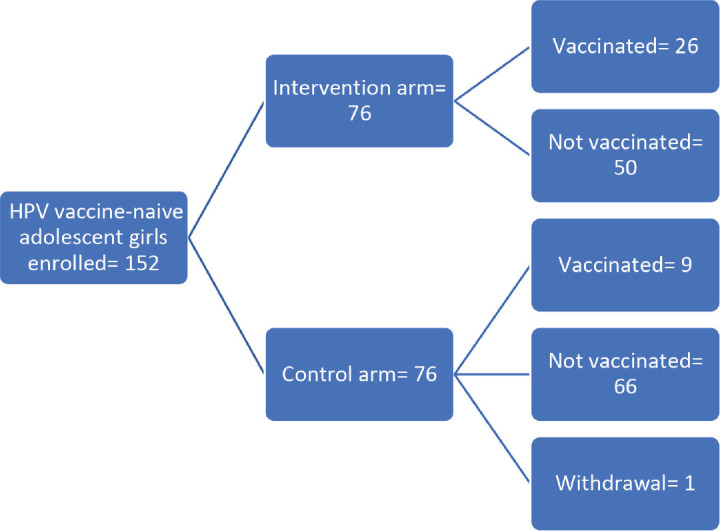
Flowchart of the study.

The study enrolled 152 HPV vaccine-naïve adolescents, of whom 151 completed the follow-up, which started on 1st August 2023 and ended on 31st January 2024. One participant in the control arm withdrew from the study during the second week of follow up. Consequently, the final analysis excluded this withdrawal (Figure 1).

There was no significant difference between the profile of participants in the control and in the intervention arms with almost half (49.3%) reporting to have never heard about HPV and majority (60.9%) having never heard about the HPV vaccine (Table 1). The majority of the respondents 97.4% in the SMS-arm and 98.7% in the non-SMS arm received between 20 and 24 SMS (Table 2).

Following the text messaging intervention, 35 (23.2%) of the respondents received at least one dose of the HPV vaccine. These were 9 (25.7%) respondents in the control arm and 26 (74.3%) in the intervention arm. This difference in HPV vaccine uptake was statistically significant (*p* = 0.001) (Table 3).

**Figure 2.**
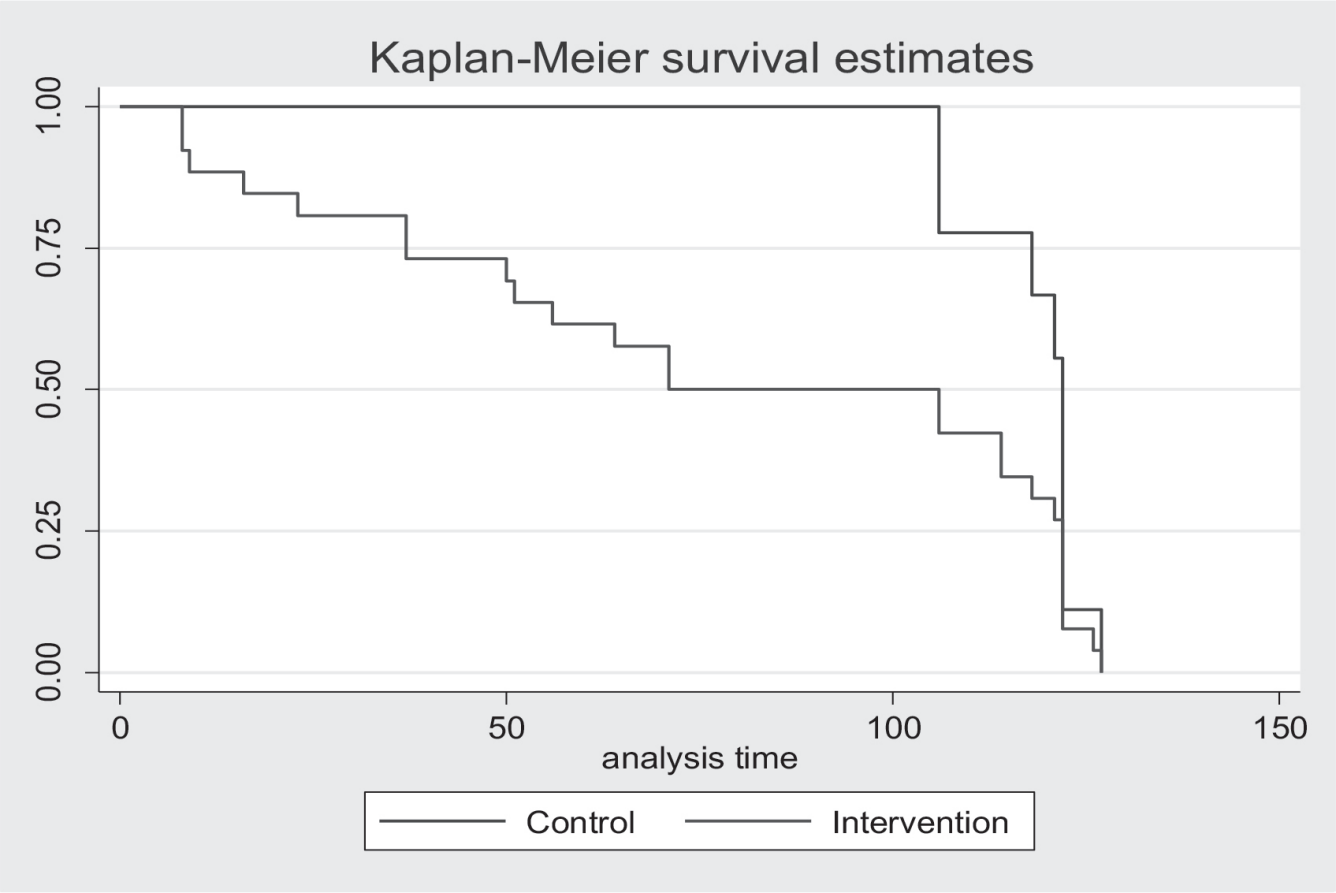
Kaplan-Meier plot of time to vaccination by arm.

Further, the Kaplan-Meier plot showed a difference in vaccination status between the non-SMS and the SMS arms (Figure 2).

## Discussion

The study enrolled vaccine-naïve adolescents majority of who had never heard about the HPV vaccine, they were either in primary or secondary level of education. There was a significant difference in the HPV vaccination rates between the intervention (34.2%) and the control (12.0%) arms following the text messaging intervention.

The use of text messaging have been used in other setting yielding similar positive results where they have been reported to be effective when addressing diabetes self-management, weight loss, physical activity, smoking cessation and medication adherence for antiretroviral therapy [28]. Similarly, a combination of text messaging and mobile social networking have also been reported to be effective in promoting physical activity and reducing anthropometric indices [29].

Receiving SMS reminders has also shown to increase participants likelihood of taking up the seasonal influenza vaccine by up-to 30% [30]. Furthermore combination of SM) with phone calls has been found to improve the rate of pentavalent vaccine completeness [31]. A review of literature highlights the impact of SMS vaccination reminders in improving vaccination metrics, including increase in vaccination coverage, decrease in dropout rates, improving completion rates and minimising vaccination delays [32].

However, our results differ from findings from other studies, such as a study that evaluated weekly text messages sent to pregnant women to reinforce the recommendation for and safety of the influenza vaccine in pregnancy, which did not increase the likelihood of actually receiving the vaccine [33]. Further, a behavioural SMS intervention aimed at improving cardiovascular health in women, although feasible and well-received, did not lead to improved health outcomes [34].

We also documented the dates of vaccination to determine the time to vaccination. The results also demonstrated a shorter time-to-vaccination in the intervention arm compared to the control arm. Similar results have been reported in the literature, for example, use of SMS vaccination reminder has led reduction in the delay for vaccination [32]. However, the study did not collect data on the number of those who declined the vaccine when offered. This highlights the potential of SMS intervention to stimulate and boost the demand for HPV vaccine.

As Kenya makes steps towards realisation of the first 90 in the WHO 90-70-90 targets of elimination of cervical cancer as a public health problem, targeted communication models will play a key role in getting the adolescent girls to vaccinate. Text messaging provides a unique opportunity for the Ministry of Health to reach this critical population with the relevant, culturally sensitive and personalised information to address the misinformation that is dragging back the uptake of the HPV vaccine.

The current study was able to demonstrate how use of SMS can improve uptake of at least once dose of HPV vaccine and shorten the decision-making time for vaccine uptake. However, the current study did not assess vaccine completion rates due to the short follow-up period. Further, delivery of the SMSs and comprehension of the messages could not be verified and only based on respondents’ verbal report. Future studies should assess vaccine completion rates, delivery of the SMSs and comprehension of the messages shared.

## Conclusion

Targeted communication using SMS is an effective channel of vaccine communication, which is required to address vaccine hesitancy and create demand for HPV vaccine. The SMS intervention can reduce the time it takes for the parents and their adolescent girls to make decision on taking up the vaccine and actually being vaccinated.

## Limitations of the study

The current study did not assess vaccine completion rate and the intention to vaccinate among those yet to get the vaccine. Future studies should consider assessing the vaccine completion rates and the intention to vaccinate among those yet to be vaccinated.

## Conflicts of interest

The authors declare no conflicts of interest.

## Consent for publication

Not applicable.

## Ethics approval and consent to participate

The study obtained ethical approval from the Moi Teaching and Referral Hospital/Moi University’s Institutional Research and Ethics Committee (REF: IREC 346/2022) and research permit issued by the National Commission for Science, Technology and Innovation (No. NACOSTI/P/23/24291). Before enrolment of the adolescent girls, written informed consent was obtained from the parent who also provided their contact details through which the text messages would be shared, and the adolescent girls gave written informed assent. For the parents, written informed consent was obtained before enrolment.

## Availability of data and materials

The datasets used and/or analysed during the current study are available from the corresponding author on reasonable request.

## Author contributions

OEO designed and carried out the data collection in the field and participated in the drafting of the manuscript. PT, KM, EOO and PI made substantial contributions to the design and interpretation of the data. SA reviewed and analysed the data. OEO, KM, SA, PT, PI, EOO, BR, PL and SC were involved in revising the manuscript critically for important intellectual content. They also gave the final approval of the version to be published and have agreed to be accountable for all aspects of this work. All authors read and approved the final manuscript.

## Figures and Tables

**Table 1.** Baseline assessment of the study participants.

Variables		Control, *n* (%)	Intervention, *n* (%)	Total, *n* (%)	*p*-value
					
Age	Mean (SD)	12.3 (1.7)	12.2 (1.4)	12.2 (1.6)	0.465
	Median (IQR)	13.0 (11.0, 14.0)	12.0 (11.0, 13.0)	13.0 (11.0, 14.0)	
					
Level of education	In primary school	32 (42.7)	40 (52.6)	73 (48.0)	0.106
	In secondary	43 (57.3)	36 (47.4)	79 (52.0)	
					
Heard about HPV	1- Yes	41 (54.7)	34 (44.7)	77 (50.7)	0.382
	0- No	34 (45.3)	42 (55.3)	75 (49.3)	
					
Heard about HPV-vaccine	1- Yes	31 (41.3)	28 (36.8)	59 (39.1)	0.576
	0- No	44 (58.7)	48 (63.2)	92 (60.9)	

**Table 2.** SMS delivery rates.

No. of SMS received	SMS-arm {*n* = 76(%)}	Non-SMS-arm {*n* = 75(%)}	*p*-value
10–14	0 (0.0)	1 (1.3)	0.709
15–19	2 (2.6)	0 (0.0)	
20–24	74 (97.4)	74 (98.7)	
Total	76 (100.0)	75 (100.0)	

**Table 3.** HPV vaccine uptake following the text messaging intervention.

Variable	Non-SMS arm, *n* (%)	SMS arm, *n* (%)	Total, *n* (%)	*p*-value
Vaccinated	9 (12.0)	26 (34.2)	35 (23.2)	**0.001**
Not vaccinated	66 (88.0)	50 (65.8)	116 (76.8)	

Level of significance was determined at *p* ≤ 0.05. Bold are statistically significant

## References

[1] GLOBOCAN (2023). Cervix Uteri Fact Sheet.

[2] Sung H, Ferlay J, Siegel RL (2021). Global cancer statistics 2020: GLOBOCAN estimates of incidence and mortality worldwide for 36 cancers in 185 countries [Internet]. CA Cancer J For Clin.

[3] Asempah E, Ikpebe E (2024). Accelerating HPV vaccination in Africa for health equity [Internet]. Glob Heal Res Policy.

[4] Asempah E, Wiktorowicz ME (2023). Understanding HPV vaccination policymaking in rwanda: a case of health prioritization and public-private-partnership in a low-resource setting [Internet]. Int J Environ Res Public Health Internet.

[5] Stelzle D, Tanaka LF, Lee KK (2021). Estimates of the global burden of cervical cancer associated with HIV [Internet]. Lancet Glob Heal.

[6] Lacey CJ (2019). HPV vaccination in HIV infection [Internet]. Papillomavirus Res (Amsterdam Netherlands).

[7] Bruni L, Saura-Lázaro A, Montoliu A (2021). HPV vaccination introduction worldwide and WHO and UNICEF estimates of national HPV immunization coverage 2010–2019. Prev Med (Baltim).

[8] Dorji T, Nopsopon T, Tamang ST (2021). Human papillomavirus vaccination uptake in low-and middle-income countries: a meta-analysis [Internet]. EClinicalMedicine Internet.

[9] Arbyn M, Xu L, Simoens C (2018). Prophylactic vaccination against human papillomaviruses to prevent cervical cancer and its precursors [Internet]. Cochrane Database Syst Rev.

[10] Brisson M, Kim JJ, Canfell K (2020). Impact of HPV vaccination and cervical screening on cervical cancer elimination: a comparative modelling analysis in 78 low-income and lower-middle-income countries [Internet]. Lancet.

[11] Wang R, Pan W, Jin L (2020). Human papillomavirus vaccine against cervical cancer: opportunity and challenge [Internet]. Cancer Lett.

[12] Spinner C, Ding L, Bernstein DI (2019). Human papillomavirus vaccine effectiveness and herd protection in young women [Internet]. Pediatrics.

[13] Pingali C, Yankey D, Elam-Evans LD (2021). National, regional, state, and selected local area vaccination coverage among adolescents aged 13–17 years - United States, 2020 [Internet]. MMWR Morb Mortal Wkly Rep Internet.

[14] Baussano I, Sayinzoga F, Tshomo U (2021). Impact of human papillomavirus vaccination, Rwanda and Bhutan [Internet]. Emerg Infect Dis.

[15] MOH (2023). Kenya National Immunization Policy Guidelines.

[16] Bruni L, Albero G, Serrano B (2021). www.hpvcentre.net.

[17] Karanja-Chege CM (2022). HPV Vaccination in Kenya: the challenges faced and strategies to increase uptake [Internet]. Front Public Heal.

[18] John SI (2021). Kenya’s HPV Vaccine Introduction and JSI’s Experiences.

[19] Shin Y, Kim SK, Lee M (2019). Mobile phone interventions to improve adolescents’ physical health: a systematic review and meta-analysis [Internet]. Public Health Nurs Internet.

[20] Mehra N, Tunje A, Hallström IK (2021). Effectiveness of mobile phone text message reminder interventions to improve adherence to antiretroviral therapy among adolescents living with HIV: a systematic review and meta-analysis. PLoS One.

[21] Feroz AS, Ali NA, Khoja A (2021). Using mobile phones to improve young people sexual and reproductive health in low and middle-income countries: a systematic review to identify barriers, facilitators, and range of mHealth solutions [Internet]. Reprod Health Internet.

[22] Levey EJ, Onyeaka H, Bartles SM (2021). Mobile technology access and use among adolescent mothers in Lima, Peru: mixed methods study [Internet]. JMIR Pediatr Parent Internet.

[23] Nabirye J, Okwi LA, Nuwematsiko R (2020). Health system factors influencing uptake of Human Papilloma Virus (HPV) vaccine among adolescent girls 9–15 years in Mbale District, Uganda [Internet]. BMC Public Health Internet.

[24] Gowda C, Schaffer SE, Dombkowski KJ (2012). Understanding attitudes toward adolescent vaccination and the decision-making dynamic among adolescents, parents and providers [Internet]. BMC Public Health Internet.

[25] Herman R, Mcnutt LA, Mehta M (2019). Vaccination perspectives among adolescents and their desired role in the decision-making process [Internet]. Hum Vaccin Immunother Internet.

[26] NASCOP (2020). Kenya HIV Estimates Report 2020.

[27] Prasad K (2020). Sample size calculation with simple math for clinical researchers [Internet]. Natl Med J India.

[28] Hall AK, Cole-Lewis H, Bernhardt JM (2015). Mobile text messaging for health: a systematic review of reviews [Internet]. Annu Rev Public Health.

[29] Ansari K, Afshari P, Abedi P (2022). Comparing the effects of text messaging and mobile social networking on physical activity and anthropometric indices of middle-aged women: a randomized controlled trial [Internet]. BMC Womens Health Internet.

[30] Esteban-Vasallo MD, Domínguez-Berjón MF, García-Riolobos C (2019). Effect of mobile phone text messaging for improving the uptake of influenza vaccination in patients with rare diseases [Internet]. Vaccine.

[31] Yunusa U, Ibrahim AH, Ladan MA (2022). Effect of mobile phone text message and call reminders in the completeness of pentavalent vaccines in Kano state, Nigeria [Internet]. J Pediatr Nurs.

[32] Manakongtreecheep K (2017). SMS-reminder for vaccination in Africa: research from published, unpublished and grey literature [Internet]. Pan Afr Med J Internet.

[33] Yudin MH, Mistry N, De Souza LR (2017). Text messages for influenza vaccination among pregnant women: a randomized controlled trial. Vaccine.

[34] Acevedo M, Varleta P, Casas-Cordero C (2023). Original research: mobile-phone text messaging to promote ideal cardiovascular health in women [Internet]. Open Heart Internet.

